# Early maladaptive schemas, coping strategies, and functional impairments in individuals with adjustment disorder during compulsory military service: a comparison with healthy controls

**DOI:** 10.3389/fpsyt.2025.1619638

**Published:** 2025-07-14

**Authors:** Tarık Sağlam

**Affiliations:** Department of Psychiatry, Erzurum City Hospital, University of Health Sciences, Erzurum, Türkiye

**Keywords:** adjustment disorder, early maladaptive schemas, coping strategies, functional impairment, military mental health, schema therapy

## Abstract

**Background:**

Adjustment Disorder (AD) is a psychological condition that arises as a response to identifiable stressors, leading to emotional distress and functional impairment. This cross-sectional study, conducted within the context of compulsory military service in Turkey, aimed to investigate the association between early maladaptive schemas (EMS), coping strategies, and functional outcomes in individuals diagnosed with AD. The study population consisted of male conscripts who developed AD symptoms following relocation and psychosocial stressors specific to the military setting, such as loss of autonomy, limited support, and institutional pressure. EMS are deep-seated cognitive patterns that shape an individuals’ responses to stress and may contribute to the development of AD. However, the relationship between EMS, coping strategies, and functional outcomes in AD remains unclear.

**Methods:**

This cross-sectional study, included 113 male participants diagnosed with Adjustment Disorder and 75 healthy male controls, aged between 18 and 40 years. The clinical group consisted of male conscripts who developed AD symptoms during their mandatory military service. The participants completed self-report measures, including the Young Schema Questionnaire (YSQ-SF3), Coping Attitudes Inventory (CAI, a self-report instrument designed to assess coping strategies), and Functionality Assessment Short Test (FAST). The groups were compared and correlations between EMS, coping strategies, and functionality were examined using statistical tools.

**Results:**

Participants with AD scored significantly higher on maladaptive schemas, particularly in failure, enmeshment/dependence, and pessimism, showed greater use of avoidance-based coping strategies, and exhibited poorer overall functioning compared to healthy controls (p <.001, η² = .072–.384). Maladaptive schemas were negatively correlated with adaptive coping (r = –.389 to –.565) and positively associated with avoidance and functional impairment (r = .573 to.734, p <.01).

**Conclusions:**

Our findings suggest that maladaptive schemas may play a key role in how individuals with AD cope with stress, often leading to avoidance behaviors and functional decline. Addressing these schemas through schema-focused therapy may help individuals develop healthier coping strategies and improve overall functioning. Further research, including randomized controlled trials evaluating schema-focused interventions, is needed to explore long-term treatment outcomes, particularly in populations exposed to institutional or occupational stressors such as compulsory military service.

## Introduction

Adjustment disorder (AD) is a psychiatric condition characterized by emotional and behavioral responses to identifiable psychosocial stressors, resulting in significant impairment in functioning (1). According to the American Psychiatric Association (2013) and the ICD-11 criteria, AD develops in response to a specific stressor and can negatively impact an individual’s functioning (1, 2). AD is also referred to as an “adjustment syndrome” and is associated with severe emotional distress (3). AD shares certain similarities with post-traumatic stress disorder (PTSD); while PTSD is typically linked to life-threatening events, AD can arise from more common stressors. Although AD and PTSD may appear similar due to their association with external stressors, they differ substantially in terms of diagnostic criteria and symptomatology. PTSD typically involves exposure to life-threatening or highly traumatic events and is marked by intrusive thoughts, flashbacks, and persistent avoidance of trauma-related stimuli. In contrast, AD results from non-traumatic but significant psychosocial stressors and is characterized by emotional or behavioral symptoms that are disproportionate to the stressor, yet insufficient to meet criteria for other specific mental disorders. Although AD is frequently diagnosed by clinicians, it has been studied less extensively compared to other psychiatric disorders (4). The stressors that lead to AD can vary between individuals (5). Paykel et al. (3) classified life events as “desirable/undesirable” (e.g., career advancement/illness) and “entry/escape” (e.g., marriage/loss of a loved one) (3). Similarly, mandatory and structured stressful life events, such as a compulsory military service, may have adverse psychological effects on individuals.

Military service constitutes a unique context for AD due to its distinct psychosocial stressors. These may include forced relocation, interpersonal conflicts with superiors, disruption of daily routines, chronic sleep disturbances, intense physical training demands, limited personal autonomy, reduced access to social support, and difficulties adapting to strict institutional hierarchies and environments. Notably, the clinical sample in our study comprised individuals who developed symptoms of AD in response to military-related stressors that, while not formally classified as traumatic, involved significant and sustained psychological strain. Therefore, our findings regarding schemas, coping strategies, and functional impairments should be interpreted within this specific institutional and psychosocial context. The response to stressors depends on individual factors, including age, gender, health status, and comorbid psychiatric conditions, as well as contextual factors, such as educational level, social support, religious beliefs, and economic conditions (6).

In this context, stress can be defined as an individual’s inherent response to a stimulus, pressure originating from an external source, or a dynamic process involving both internal and external factors (7, 8). Coping strategies play a critical role in maintaining psychological well-being and are associated with dynamic, cognitive, and behavioral efforts by the individual (9). Research on the clinical course of AD primarily focuses on two key areas: consultation-liaison psychiatry and military mental health. Within the realm of consultation-liaison psychiatry, AD may arise in association with chronic illnesses or highly stressful conditions and can predispose individuals to other psychiatric disorders (10). Military personnel represent a distinct at-risk group for AD due to their continuous exposure to stress. A study conducted in the United States found that AD was implicated in one-third of newly diagnosed mental health disorders among military personnel and that early diagnosis could influence the duration of a military career (11). The progression of AD, including whether the disorder becomes chronic or evolves into more severe psychiatric conditions, is a critical research area in both military and civilian populations due to its impact on functional impairment (11, 12).

Early maladaptive schemas (EMS) are stable cognitive-emotional frameworks, developed during childhood and adolescence, that profoundly affect individuals’ interpretations and responses to stress (13). While extensive research has focused on EMS in relation to chronic psychopathologies, particularly personality disorders and persistent mood/anxiety conditions (14), their role in acute stress-related disorders, such as AD, remains less explored. Investigating EMS in AD represents a novel and significant contribution to the literature, as it offers insights into schema-driven vulnerabilities that shape coping mechanisms and contribute to functional impairments, potentially improving therapeutic outcomes through targeted schema-based interventions. EMS may represent a key mechanism in the development of AD. According to Young et al. (13), EMS are persistent cognitive and emotional patterns that develop through early life experiences and influence how individuals perceive, interpret, and regulate their environment. EMS are therefore critical factors that can shape the stress-coping mechanisms of an individual and are likely to play a significant role in stress-related psychiatric disorders such as AD (15). To the best of our knowledge, studies directly examining the relationship between AD and EMS are limited.

Individuals may develop various strategies to manage the activation or potential activation of EMS. According to Young’s conceptualization, three primary coping mechanisms are utilized as a response to EMS: surrender, avoidance, and overcompensation (16). Surrender entails the acceptance of the schema as true and behaving accordingly. Avoidance refers to cognitive or behavioral strategies aimed at preventing the activation of the schema, such as emotional detachment or distraction. Overcompensation, on the other hand, is characterized by an attempt to counteract the schema by adopting exaggeratedly opposite beliefs and behaviors. It is important to note that these coping mechanisms primarily serve to manage internal emotional and cognitive experiences rather than external circumstances. Additionally, these responses are often automatic, occurring without conscious awareness (16). Understanding these coping patterns is therefore essential to evaluate the persistence of maladaptive schemas and their role in psychological distress.

Given the limited number of studies directly addressing the relationship between AD and EMS, the present study seeks to determine whether EMS represent a risk factor in the development and persistence of AD symptoms.

## Research Hypotheses

Based on the existing literature and clinical observations, this study hypothesizes that individuals diagnosed with Adjustment Disorder would report higher levels of maladaptive schemas—particularly Failure, Enmeshment/Dependence, Pessimism, Entitlement/Insufficient Self-Control, and Social Isolation/Mistrust—along with increased use of avoidance-based coping strategies and impaired functionality, compared to healthy controls. The rationale behind focusing on schemas, coping, and functionality in individuals with AD is to identify underlying cognitive-emotional vulnerabilities (schemas) that shape maladaptive coping responses. While the primary analyses are hypothesis-driven, secondary exploratory analyses were also conducted to examine additional associations that could further clarify the clinical picture of AD.

## Materials and methods

### Study design and participants

This cross-sectional, scale-based study was conducted between February 1, 2023, and February 15, 2025. The clinical group consisted of male individuals who presented to the psychiatry outpatient clinic at the [Name] University Faculty of Medicine Research Hospital with complaints of adjustment difficulties, irritability, and insomnia following their mandatory service placement at a new, male-only institution. The control group comprised healthy male volunteers who applied to the hospital’s medical reporting unit for routine check-ups and had no history of psychiatric complaints or diagnoses.

An *a priori* power analysis was conducted using the G*Power software to determine the appropriate sample size. Assuming a large effect size (Cohen’s d = 0.80), a significance level of α = 0.05 (two-tailed), and desired statistical power of 0.95, the minimum required sample size per group was calculated to be 42 participants. The final sample size of 113 participants in the clinical group and 75 controls exceeded this minimum requirement, ensuring adequate power for detecting the hypothesized effects. This assumption was informed by previous research demonstrating large differences in maladaptive schema scores between clinical and non-clinical populations (17).

A total of 113 individuals diagnosed with AD and 75 control participants were included. All participants were recruited consecutively, and written informed consent was obtained prior to participation. The sample consisted exclusively of male participants, as the data were collected within a compulsory military service environment in Turkey that included only male conscripts at the time of the study. Additionally, gender may influence stress responses and coping strategies; thus, using a gender-homogeneous sample allowed for more controlled interpretation of EMS and coping relationships in this specific psychosocial context.

In Turkey, compulsory military service is mandated for all male citizens between the ages of 20 and 41. The process is centrally coordinated by the Ministry of National Defense. Eligibility is determined through a standardized evaluation system that includes both medical and psychological screening. Individuals with chronic physical illnesses, psychiatric disorders, or intellectual disabilities are exempted from service. Those who are deemed fit are assigned to military units across the country, often with limited control over location and role. This system may impose significant psychosocial stressors such as relocation, loss of autonomy, and reduced access to social support, which are relevant to the onset of adjustment-related symptoms.

Inclusion criteria for the clinical group were: male sex, age between 18 and 40 years, diagnosis of AD according to DSM-5 criteria confirmed through clinical evaluation and the Structured Clinical Interview for DSM-5 (SCID-5), administered by two experienced psychiatrists. Participants were excluded if they had a history of neurological illness, head trauma, or substance/alcohol use within the past six months, or if they had any current or past psychiatric diagnoses other than AD.

The control group included age-matched male individuals who underwent structured clinical interviews by trained psychiatrists to rule out any current or past psychiatric diagnoses, as well as neurological or substance use disorders. Control participants were confirmed to be free of psychiatric conditions based on the SCID-5 interview. To ensure diagnostic homogeneity, all participants were assessed using the SCID-5 by trained psychiatrists. Individuals with PTSD or any other Axis I psychiatric disorders were excluded during this process. Exclusion criteria for both groups included intellectual disability, illiteracy, and the presence of psychiatric comorbidities such as mood, anxiety, psychotic, personality, or substance use disorders, as evaluated in accordance with DSM-5 diagnostic criteria.

All participants were informed about the study’s aims and procedures and voluntarily agreed to participate. No individual refused participation, and no monetary or other incentives were provided.

Ethical approval was obtained from the university’s local ethics committee on February 28, 2024. This study was reviewed and approved by the Erzurum City Hospital Ethics Committee (Date: 15.02.2024, Approval no: B.30.2.ATA.0.01.00/161). All participants provided written informed consent prior to participation. Data were collected through face-to-face interviews conducted by trained psychiatrists. All participants who met the study criteria completed the Sociodemographic Data Form, the Young Schema Questionnaire – Short Form 3 (YSQ-SF3), the Coping Attitudes Inventory (CAI), and the Functioning Assessment Short Test (FAST). All scales were self-reported. Data were anonymized and securely stored.

A *post hoc* power analysis indicated that the sample size was sufficient to detect large between-group effect sizes (Cohen’s d ≥ 0.80) with a power of 0.999 and alpha = 0.05 (two-tailed), confirming the adequacy of the study’s statistical power.

### Procedure

#### Instruments and measurements

##### Sociodemographic data form

This form queried information on age, gender, educational status, occupation, employment status, marital status, smoking-alcohol-substance use, history of traumatic experiences, parental relationship status, and past suicide attempts of the participants.

##### The young schema questionnaire-short form 3

The YSQ-S3 (Young, 2005) consists of 90 items designed to assess 18 maladaptive schemas (18). The participants rate each item on a six-point Likert scale, ranging from 1 (completely untrue) to 6 (completely true). Each schema is represented by five items, with total scores varying between 5 and 30. A sample statement from the YSQ-S3 is as follows: “I often feel that others may take advantage of me,” which falls under the mistrust/abuse subscale. Previous studies have reported a satisfactory test-retest reliability (19) and strong internal consistency across both clinical and non-clinical populations (20). In the present study, the internal consistency of the overall scale, measured by Cronbach’s alpha, was.99, while the subscale reliability coefficients ranged from.72 to.93.

##### Coping attitudes inventory

The CAI was used to assess coping strategies for stress. The original inventory was adapted to the Turkish by Özbay (21). The CAI consists of 43 items and six subscales and assesses an individuals’ coping efforts when facing various stressful events. The subscales include active planning, seeking external support, turning to religion as a means of coping, emotional disengagement/avoidance, biochemical disengagement/avoidance, and acceptance-cognitive restructuring. The scale follows a 5-point Likert format, with possible scores ranging from 23 to 115. The Cronbach’s alpha reliability coefficient of the scale was determined to be 0.81, while the general reliability analysis in this study yielded a Cronbach’s alpha of 0.76. Internal consistency (Cronbach’s alpha) values for the CAI subscales in the current sample were as follows: Active Planning (α = .76), Seeking External Support (α = .81), Turning to Religion (α = .73), Escape-Avoidance (Emotional-Behavioral) (α = .78), Escape-Avoidance (Biochemical) (α = .75), and Acceptance-Cognitive Restructuring (α = .82).

##### The functioning assessment short test

The FAST was developed by Rosa et al. (22) and adapted to the Turkish by Aydemir and Uykur (23). The FAST scale was designed to quickly and practically assess functional impairment related to psychiatric disorders. The scale consists of 24 items and is categorized into six subdomains: autonomy, occupational functionality, cognitive functionality, financial matters, interpersonal relationships, and leisure activities. Each item is scored on a Likert scale ranging from 0 (no difficulty) to 3 (severe difficulty), with the total score calculated by summing all item scores, where higher scores indicate greater functional impairment. The Cronbach’s alpha reliability coefficient of the original scale was reported as 0.960, while in this study, the reliability coefficient was found to be 0.98.

### Statistical analysis

All statistical analyses were conducted using IBM SPSS Statistics version 23.0 (IBM Corp., Armonk, NY, USA). Descriptive statistics were calculated to summarize demographic, clinical, and psychological characteristics. Continuous variables were presented as means, standard deviations, and categorical variables as frequencies and percentages. Group comparisons for continuous variables were performed using independent samples t-tests, while Chi-square (χ²) tests and Likelihood Ratio tests were applied for categorical variables, depending on the data distribution. Where appropriate, effect sizes were reported using Cohen’s d for continuous variables and Cramér’s V for categorical comparisons. To assess differences in maladaptive schemas and stress coping strategies between groups while controlling potential covariates (e.g., age, education, work status, parental situation), Analysis of Covariance (ANCOVA) was used. F-values, p-values, and partial eta squared (η²) values were reported to evaluate the significance and strength of effects. Partial eta squared (η²) values were interpreted according to Cohen’s guidelines: 0.01 indicates a small effect size, 0.06 indicates a medium effect size, and values of 0.14 or higher indicate a large effect size. Additionally, Pearson correlation analyses were performed to explore relationships between maladaptive schemas, coping strategies, and functional impairment. A two-tailed significance level of p <.05 was considered statistically significant for all analyses.

### Ethical approval

Ethical approval: The research protocol has been reviewed and approved by Erzurum City Hospital Ethics Committee [B.30.21.ATA.0.01.00/161], strictly adhering to the principles outlined in the Declaration of Helsinki.

## Results

### Demographic and clinical characteristics

**Table 1** presents the demographic and clinical characteristics of individuals with AD and healthy controls. The mean age in the AD group was significantly lower than that of the healthy control group (22.46 ± 2.60 vs. 29.28 ± 5.69; t = –11.107, p <.001, Cohen’s d = 1.65). Similarly, the average number of years of education was significantly lower in the AD group (9.27 ± 2.28) compared to the control group (14.81 ± 2.79; t = –14.837, p <.001, Cohen’s d = 2.22). No significant difference was found in employment status between the two groups (p = .272, Cramér’s V = 0.118).

**Table 1:** Demographic and clinical characteristics of participants with adjustment disorders and healthy controls.

Variables	Groups		t	*p*	*Cramér's V /Cohen’s d*
	Adjustment disorders (n=113)	Healthy Control (n=74)			
	Mean ± SD	Mean ± SD			
Age**	22.46 ± 2.60	29.28 ± 5.69	-11.107	<.001	1.65
Education levels**	9.27 ± 2.28	14.61 ± 3.25	-13.333	*<.001*	*2.22*
	n (%)	n (%)	χ2	*p*	
Working status*			2.607	.272	0.118
Unemployed or irregular work	5 (4.4)	6 (8.0))			
Regularly work	28 (34.6)	67 (82.7)			
Smoker (yes)*	104 (92.0)	36 (48.0)	45.976	<.001	0.495
Alcohol Consumption*			101.689	<.001	0.735
Does not consume	9 (8.0) ^a^	54 (72.0) ^b^			
Occasionally consumes	34 (30.1) ^a^	21 (28.0) ^a^			
Consumes regularly	70 (61.9) ^a^	0 (0.0) ^b^			
Substance Use*			119.580	<.001	0.798
Does not use	21 (18.6) ^a^	75 (0.0) ^b^			
Former user	30 (26.5) ^a^	0 (0.0) ^b^			
Current user	62 (54.9) ^a^	0 (0.0) ^b^			
History of self-mutilation (yes)*	78 (69.0)	3 (4.0)	77.734	<.001	0.643
History of suicide attempts *	21 (18.6)	1 (1.3)	12.983	<.001	0.263
Parental situation***			43.727	<.001	0.435
Living together	60 (53.1) ^a^	68 (90.7) ^b^			
Divorced/separated	46 (40.7) ^a^	2 (2.7) ^b^			
Mother deceased	3 (2.7) ^a^	1 (1.3) ^a^			
Father deceased	4 (3.5) ^a^	4 (5.3) ^a^			

p <0.05 statistically significant (bold values). *Pearson Chi-Square Test, **Student's t-test, ***Likelihood Ratio Test. used; χ2, Chi-square; SD, Standard deviation.

The superscript letters “a” and “b” indicate statistically significant differences between the Adjustment Disorder group (a) and the Healthy Control group (b).

Regarding lifestyle factors, the AD group showed significantly higher rates of smoking (92.0% vs. 48.0%; χ² = 45.976, p <.001, Cramér’s V = 0.495), regular alcohol consumption (61.9% vs. 0.0%; χ² = 101.689, p <.001, Cramér’s V = 0.735), and substance use (current use: 54.9% vs. 0.0%; χ² = 119.580, p <.001, Cramér’s V = 0.798) compared to healthy controls. Additionally, individuals with AD reported significantly more frequent histories of self-mutilation (69.0% vs. 4.0%; χ² = 77.734, p <.001, Cramér’s V = 0.643) and suicide attempts (18.6% vs. 1.3%; χ² = 12.983, p <.001, Cramér’s V = 0.263). Parental marital status also differed significantly between the groups, with a higher proportion of divorced or separated parents reported in the AD group (40.7% vs. 2.7%; χ² = 43.727, p <.001, Cramér’s V = 0.435).

### Young schema questionnaire scores

As shown in **Table 2**, participants with AD scored significantly higher than healthy controls on nearly all subscales of the Young Schema Questionnaire – Short Form Version 3 (YSQ-SF3). The most prominent differences were observed in the failure schema (22.23 ± 6.98 vs. 7.36 ± 2.47; F = 111.744, p <.001, η² = .384), enmeshment/dependence (30.04 ± 8.44 vs. 10.36 ± 3.85; F = 83.946, p <.001, η² = .319), and pessimism (22.64 ± 6.23 vs. 7.80 ± 3.96; F = 73.109, p <.001, η² = .290). Other schema domains, such as defectiveness, social isolation/mistrust, emotional deprivation, and abandonment, also showed large and statistically significant differences between groups (all p <.001, η² ranging from.201 to.263). The only subscale that did not differ significantly between groups was high standards (F = 1.776, p = .184, η² = .010).

**Table 2:** Comparison of maladaptive schema scores (YSQ-SF3) between adjustment disorders and healthy controls.

YSQ-SF3 Subscales	Groups		F	*p*	*Partial eta squared (η²)*
	Adjustment Disorders (n=113)	Healthy Control (n=74)			
	Mean ± SD	Mean ± SD			
*Emotional Deprivation*	17.04 ± 4.56	6.81 ± 2.85	647.091	.000	.205
*Failure*	22.23 ± 6.98	7.36 ± 2.47	111.744	.000	.384
*Pessimism*	22.64 ± 6.23	7.80 ± 3.96	73.109	.000	.290
*Social isolation/Mistrust*	32.65 ± 8.88	10.39 ± 5.74	53.616	.000	.230
*Emotional Inhibition*	19.37 ± 5.27	8.47 ± 4.14	32.480	.000	.154
*Approval-Seeking*	22.71 ± 6.75	13.92 ± 5.84	23.104	.000	.114
*Enmeshment/Dependence*	30.04 ± 8.44	10.36 ± 3.85	83.946	.000	.319
*Entitlement/Insufficient Self- Control*	31.63 ± 8.87	15.21 ± 6.79	25.567	.000	.125
*Self-Sacrifice*	19.04 ± 5.38	11.48 ± 4.94	13.846	.000	.072
*Abandonment*	17.14 ± 7.02	6.61 ± 3.07	45.128	.000	.201
*Punitiveness*	22.07 ± 7.63	15.55 ± 5.91	16.671	.000	.085
*Defectiveness*	22.52 ± 6.47	7.48 ± 3.35	63.951	.000	.263
*Vulnerability to Harm*	19.15 ± 6.33	7.08 ± 3.67	40.436	.000	.184
*High Standards*	10.35 ± 4.24	7.91 ± 4.03	1.776	.184	.010

ANCOVA was conducted to compare maladaptive schema scores between groups while controlling for age, years of education, smoking status, alcohol consumption, substance use, and parental situation. p <.05 was considered statistically significant. Mean, Mean; SD, Standard deviation; YSQ-SF3, Young's Schema Questionnaire – Short Form Version 3.

### Stress coping strategies

**Table 3** shows group differences in stress coping strategies and functional outcomes. Individuals with AD demonstrated significantly higher use of escape-avoidance coping strategies, including both emotional-behavioral (12.77 ± 5.50 vs. 8.00 ± 4.86; F = 22.125, p <.001, η² = .110) and biochemical avoidance (8.13 ± 3.07 vs. 1.95 ± 2.25; F = 28.684, p <.001, η² = .138), compared to healthy controls. Additionally, the AD group showed significantly greater functional impairment, as measured by the Functioning Assessment Short Test (FAST) (33.94 ± 13.07 vs. 6.77 ± 10.97; F = 14.253, p <.001, η² = .075). In contrast, adaptive coping strategies such as active planning, seeking external support, turning to religion, and acceptance-cognitive restructuring did not show statistically significant differences between groups (p >.05).

**Table 3:** Stress coping strategies and functional assessment in adjustment disorders and healthy controls.

Stress Coping Scale	Groups		F	*p*	*Partial eta squared (η²)*
	Adjustment Disorders (n=113)	Healthy Control (n=74)			
	Mean ± SD	Mean ± SD			
*Active Planning*	21.71 ± 5.82	26.07 ± 7.42	1.967	.162	.011
*Seeking External Support*	11.20 ± 9.29	19.95 ± 6.58	.085	.770	.005
*Turning to Religion*	9.45 ± 9.32	10.67 ± 5.47	2.471	.118	.014
*Escape-Avoidance (Emotional-Behavioral)*	12.77 ± 5.50	8.00 ± 4.86	22.125	.000	.110
*Escape-Avoidance (Biochemical)*	8.13 ± 3.07	1.95 ± 2.25	28.684	.000	.138
*Acceptance-Cognitive Restructuring*	10.70 ± 5.37	12.65 ± 4.31	.048	.827	.000
Functioning Assessment Short Test	33.94 ± 13.07	6.77 ± 10.97	14.253	.000	.075

ANCOVA was conducted to compare coping strategy subscale scores and FAST scores between groups while controlling for age, years of education, smoking status, alcohol consumption, substance use, and parental situation as covariates. p <.05 was considered statistically significant.

### Correlations between maladaptive schemas and coping strategies

**Table 4** provides detailed correlation coefficients between the YSQ-SF3 subscales and the Stress Coping Scale dimensions. Maladaptive schemas were generally negatively correlated with adaptive coping strategies such as active planning (*r* = -0.389 to -0.437, *p*<.01) and seeking external support (*r* = -0.245 to -0.565, *p*<.01). For example, emotional deprivation showed the strongest negative correlation with seeking external support (*r* = -0.565, *p*<.01), indicating that participants with heightened emotional deprivation were less likely to seek help.

**Table 4:** Correlations between maladaptive schemas and stress coping strategies.

YSQ-SF3 r	Stress Coping Scale						
	*Active Planning*	*Seeking External Support*	*Turning to Religion*	*Escape-Avoidance (Emotional-Behavioral)*	*Escape-Avoidance (Biochemical)*	*Acceptance-Cognitive Restructuring*	*Functioning Assessment Short Test*
*Emotional Deprivation*	-0.389**	-0.565**	-0.190**	0.266**	0.626**	-0.253**	0.725**
*Failure*	-0.418**	-0.418**	-0.034	0.334**	0.538**	-0.286**	0.569**
*Pessimism*	-0.382**	-0.437**	-0.130	0.293**	0.642**	-0.189**	0.734**
*Social isolation/Mistrust*	-0.407**	-0.435**	-0.111	0.325**	0.614**	-0.218**	0.641**
*Emotional Inhibition*	-0.437**	-0.432**	-0.083	0.303**	0.577**	-0.288**	0.601**
*Approval-Seeking*	-0.265*	-0.245**	-0.004	0.400**	0.431**	-0.134	0.439**
*Enmeshment/Dependence*	-0.323**	-0.375**	-0.026	0.393**	0.591**	-0.160*	0.628**
*Entitlement/Insufficient Self-Control*	-0.246**	-0.414**	-0.191**	0.325**	0.655**	-0.212*	0.644**
*Self-Sacrifice*	-0.341**	-0.291**	0.045	0.238**	0.422**	-0.133	0.478**
*Abandonment*	-0.280*	-0.313**	-0.090	0.396**	0.593**	-0.157*	0.584**
*Punitiveness*	-0.084	-0.015	0.183*	0.243**	0.201*	0.036	0.244*
*Defectiveness*	-0.391**	-0.494**	-0.116	0.332**	0.681**	-0.344**	0.699**
*Vulnerability to Harm*	-0.318**	-0.414**	-0.141	0.371**	0.602**	-0.235*	0.573**
*High Standards*	-0.055	-0.143*	0.024	0.153*	0.203**	0.059	0.336**
*Functioning Assessment Short Test*	-.424**	-.486**	-.227**	.179*	.699**	-.314**	1

r, Pearson correlation coefficient; YSQ-SF3, Young Schema Inventory-Short Form-3.

*The correlation is significant (two-tailed) at the 0.05 level.

** Correlation is significant at the 0.01 level (two-tailed).

Significant positive correlations were found between maladaptive schemas and avoidance-based coping strategies. Notably, entitlement/insufficient self-control exhibited the strongest positive correlation with biochemical avoidance (*r* = 0.655, *p*<.01), followed by defectiveness (*r* = 0.681, *p*<.01). Similarly, escape-avoidance behavioral strategies were positively associated with domains such as abandonment (*r* = 0.396, *p*<.01) and defectiveness (*r* = 0.332, *p*<.01).

The FAST scale scores were positively correlated with most maladaptive schemas, including pessimism (*r* = 0.734, *p*<.01) and defectiveness (*r* = 0.699, *p*<.01), suggesting worse functional outcomes in individuals with stronger EMS. Adaptive coping strategies such as active planning and acceptance-cognitive restructuring, however, showed negative correlations with the FAST score (*r* = -0.424 to -0.314, *p*<.01), implying better functionality in individuals employing these strategies.

## Discussion

The current study is among the very few that have examined the relationship between AD, EMS, and coping mechanisms in individuals who were committed to work at a new institution. The key finding of the current study is that individuals with AD show significantly higher scores in the maladaptive schemas of social isolation/mistrust, enmeshment/dependence, and entitlement/insufficient self-control compared to healthy controls. These schemas are strongly linked to avoidance-based coping strategies and worse functional outcomes. To our knowledge, the current study is the first to explore these specific schema patterns in individuals with AD. Given the limited research in this area, our findings can contribute to a deeper understanding of functional impairment and maladaptive coping mechanisms in this patient population.

EMS have been widely investigated across a broad spectrum of psychiatric disorders, consistently demonstrating elevated levels in clinical populations. Numerous studies have established strong associations between EMS and conditions such as major depressive disorder (24), borderline personality disorder (25), obsessive-compulsive disorder (26), PTSD (27), and eating disorders (28). For instance, individuals with borderline personality disorders tend to score high across nearly all EMS domains, particularly abandonment, emotional deprivation, and mistrust/abuse, while individuals with depression exhibit heightened defectiveness/shame, failure, and social isolation schemas (29). Similarly, patients with PTSD display elevated levels of emotional inhibition, vulnerability to harm, and mistrust/abuse schemas, while patients with obsessive-compulsive disorder often display perfectionism/unrelenting standards and emotional inhibition (30). Despite the extensive research on EMS across various psychiatric conditions, AD remains significantly understudied in this context. To address this gap, our study provides novel insights into EMS patterns in individuals with AD, revealing significantly higher scores compared to healthy controls. The most pronounced effects were observed in the schemas related to social isolation/mistrust, enmeshment/dependence, and entitlement/insufficient self-control. These findings suggest that much like other psychiatric disorders, AD may also be characterized by maladaptive cognitive and emotional patterns, further underscoring the importance of schema-focused interventions in this patient population (31). Similar schema-based mechanisms have also been documented in other clinical populations. For example, Parlak et al. (32) found that individuals with alcohol use disorder who scored high in maladaptive schema domains such as disconnection, impaired autonomy, and insufficient self-control were more likely to rely on avoidance-based coping styles and experience higher craving intensity. These findings further support the transdiagnostic relevance of EMS and the clinical importance of addressing maladaptive coping early in treatment planning (32).

Maladaptive schemas are believed to develop in response to negative parental attitudes and early traumatic experiences (32, 33). The enmeshment and dependence schema, in particular, is characterized by a lack of self-sufficiency, difficulties in making independent decisions, and a persistent reliance on others. The heightened presence of this schema in individuals with AD may reflect underlying dependent personality traits, reduced autonomy, and difficulties in emotional regulation. Barber and Harmon (34) demonstrated that overcontrolling or intrusive parental attitudes can reinforce feelings of dependence and inadequacy, potentially shaping cognitive frameworks that contribute to AD (34). In the current study, individuals with AD exhibited a decreased tendency to engage in active problem-solving strategies such as planning and seeking external support, preferring to rely more heavily on avoidance-based coping mechanisms, including emotional-behavioral and biochemical avoidance. This preference for avoidance may serve as a short-term attempt to reduce distress but is likely to reinforce maladaptive patterns, further entrenching emotional and functional impairment. The prominence of enmeshment/dependence and low self-efficacy schemas suggests that these cognitive structures not only shape emotional responses but also directly influence coping styles, potentially exacerbating AD symptoms over time and limiting adaptive stress management.

One of the most compelling insights from our study is the identification of a direct relationship between maladaptive schemas and dysfunctional coping behaviors in AD. In particular, we found that the entitlement/impaired self-control schema exhibited the strongest positive correlation with biochemical avoidance, suggesting that well beyond being merely elevated, EMS may actively shape avoidance-based coping strategies. Sakulsriprasert et al. (33) reported that the impaired limits schema domain is closely linked to difficulties in impulse regulation, which can contribute to dysfunctional behavior patterns (33). Our findings extend this perspective by demonstrating that the entitlement/impaired self-control EMS was not only prevalent in AD but also played a significant role in reinforcing biochemical avoidance, a maladaptive strategy aimed at temporary distress relief. This highlights a potential mechanism through which maladaptive schemas contribute to the clinical manifestations of AD. While elevated EMS have been observed across various psychiatric disorders, their role in influencing avoidance-based coping strategies may be particularly crucial in understanding the functional impairment seen in AD.

The impact of AD on daily functioning is a critical yet often overlooked aspect in published studies. In our study, individuals with AD exhibited significantly lower functionality, underscoring the profound disruptions this condition imposes on various domains of life. Notably, functional impairment was strongly correlated with pessimism and incompetence schemas, suggesting that deep-seated maladaptive cognitive patterns may contribute to the persistence of functional difficulties. These findings align with data from Catalina-Romero et al. (5), which indicated that AD is not merely a transient response to stressors but a condition that can lead to long-term impairment in psychosocial and occupational domains (5). Furthermore, our study highlights that individuals with AD predominantly rely on avoidance-based coping strategies, which may exacerbate functional impairment. Unlike previous studies, which have primarily focused on the association between AD and other psychological conditions, our findings offer a more nuanced understanding of how EMS can shape maladaptive coping mechanisms in AD, providing new insights into its cognitive and emotional underpinnings.

EMS can play a crucial role in shaping stress coping styles, particularly in individuals diagnosed with AD. However, the application of this schema-driven coping model specifically to AD has not been previously examined, highlighting the novelty of the current study. Given that avoidance strategies are associated with worse psychosocial functioning and increased vulnerability to substance use as a coping mechanism, our findings underscore the clinical relevance of addressing EMS while designing therapeutic interventions for AD.

Cudo et al. (35) reported that the relationship between EMS and problematic video gaming was not mediated by depression and anxiety. This suggests that certain EMS may be directly associated with symptoms of AD, rather than via the intermediary mechanisms of mood disturbances. Moreover, the significantly higher prevalence of tobacco, alcohol, and substance use in the AD group aligns with prior research indicating that such individuals often rely on avoidance-based coping strategies. McCarthy et al. (7) highlighted that substance use is commonly employed as a mechanism to mitigate stress, further reinforcing the maladaptive patterns observed in this population. This underscores the importance of addressing substance use within the therapeutic framework of AD, particularly in interventions targeting avoidance-based coping mechanisms. Furthermore, our study shows that individuals with AD tend to be younger and have lower educational attainment compared to healthy controls, a pattern that has been consistently reported in the literature. Catalina-Romero et al. (5) demonstrated that AD patients frequently come from socioeconomically disadvantaged backgrounds. These findings reinforce the notion that AD is shaped not only by cognitive and emotional factors but also by broader social determinants, necessitating a more comprehensive and integrative approach in both research and clinical practice.

Collectively, our findings provide new insights into the interplay between maladaptive schemas, avoidance-based coping strategies, and key demographic variables in AD. While our study contributes to a growing body of evidence on schema-related mechanisms in AD, further longitudinal research is needed to clarify the causal pathways and to explore potential interventions that can promote adaptive coping strategies in this patient population.

Several limitations should be acknowledged. First, the inclusion of participants solely from a male-only institution limits the generalizability to broader populations; future research should involve mixed-gender samples. Second, although structured clinical interviews were used, undetected subclinical psychiatric symptoms in the control group cannot be completely excluded. Third, the cross-sectional design precludes establishing causal relationships, and longitudinal studies are warranted. Lastly, reliance on self-report measures may introduce various forms of bias, including recall bias, social desirability bias, and subjective interpretation of questionnaire items. This suggests the need for clinician-rated or objective assessments in future research to enhance validity and reduce measurement error.

## Conclusion

The current study highlights the significant role of EMS in shaping stress coping strategies and functional outcomes in individuals diagnosed with AD. While EMS have been extensively studied in other psychopathological conditions, their specific impact on AD remains underexplored. Our study provides, for the first time, evidence that maladaptive schemas are associated with a decreased tendency to engage in adaptive coping mechanisms and an increased reliance on avoidance-based strategies, which in turn can negatively affect overall functioning. These findings emphasize the importance of integrating schema-focused therapeutic approaches, such as schema therapy and cognitive restructuring, into treatment protocols for AD. Additionally, future research should consider longitudinal studies or randomized controlled trials to further investigate the effectiveness of schema-focused interventions in improving coping strategies and functionality in AD.

## Data Availability

The raw data supporting the conclusions of this article will be made available by the authors, without undue reservation.
